# Cancer, Fertility and Me: Developing and Testing a Novel Fertility Preservation Patient Decision Aid to Support Women at Risk of Losing Their Fertility Because of Cancer Treatment

**DOI:** 10.3389/fonc.2022.896939

**Published:** 2022-06-30

**Authors:** Georgina L. Jones, Rachael H. Moss, Frances Darby, Neda Mahmoodi, Bob Phillips, Jane Hughes, Katharina S. Vogt, Diana M. Greenfield, Grete Brauten-Smith, Jacqui Gath, Tonia Campbell, Daniel Stark, Galina Velikova, John A. Snowden, Ellissa Baskind, Mariano Mascerenhas, Daniel Yeomanson, Jonathan Skull, Sheila Lane, Hilary L. Bekker, Richard A. Anderson

**Affiliations:** ^1^ Leeds School of Social Sciences, Leeds Beckett University, Leeds, United Kingdom; ^2^ Bradford Institute for Health Research, Bradford Teaching Hospitals NHS Foundation Trust, Bradford, United Kingdom; ^3^ Hull-York Medical School and Centre for Reviews and Dissemination, University of York, York, United Kingdom; ^4^ School of Health and Related Research, University of Sheffield, Sheffield, United Kingdom; ^5^ Department of Oncology and Metabolism, University of Sheffield and Sheffield Teaching Hospitals NHS Trust, Sheffield, United Kingdom; ^6^ Breast Cancer Now, London, United Kingdom; ^7^ Independent Cancer Patients’ Voice, London, United Kingdom; ^8^ Independent Researcher, Largs, United Kingdom; ^9^ Leeds Institute for Medical Research, University of Leeds, Leeds, United Kingdom; ^10^ Leeds Fertility, Leeds Teaching Hospitals NHS Trust, Leeds, United Kingdom; ^11^ Haematology and Oncology, Sheffield Children’s Hospital, Sheffield, United Kingdom; ^12^ Department of Paediatric Oncology, Oxford University Hospitals NHS Foundation Trust, Oxford, United Kingdom; ^13^ Leeds Unit of Complex Intervention Development (LUCID), School of Medicine, University of Leeds, Leeds, United Kingdom; ^14^ Research Centre for Patient Involvement (ResCenPI) Central Region Denmark, Department of Public Health, Aarhus University, Aarhus, Denmark; ^15^ MRC Centre for Reproductive Health, University of Edinburgh, Edinburgh, United Kingdom

**Keywords:** patient decision aid, fertility preservation, cancer, women, mixed-method study, survivorship, gonadotoxic treatment

## Abstract

**Background:**

Women with a new cancer diagnosis face complex decisions about interventions aiming to preserve their fertility. Decision aids are more effective in supporting decision making than traditional information provision. We describe the development and field testing of a novel patient decision aid designed to support women to make fertility preservation treatment decisions around cancer diagnosis.

**Methods:**

A prospective, mixed-method, three stage study involving: 1) co-development of the resource in collaboration with a multi-disciplinary group of key stakeholders including oncology and fertility healthcare professionals and patient partners (n=24), 2) *alpha* testing with a group of cancer patients who had faced a fertility preservation treatment decision in the past (n=11), and oncology and fertility healthcare professionals and stakeholders (n=14) and, 3) *beta* testing with women in routine care who had received a recent diagnosis of cancer and were facing a fertility preservation treatment decision (n=41) and their oncology and fertility healthcare professionals (n=3). Ten service users recruited from a closed Breast Cancer Now Facebook group and the support group Cancer and Fertility UK also provided feedback on CFM *via* an online survey.

**Results:**

A 60-page paper prototype of the Cancer, Fertility and Me patient decision aid was initially developed. Alpha testing of the resource found that overall, it was acceptable to cancer patients, healthcare professionals and key stakeholders and it was considered a useful resource to support fertility preservation treatment decision-making. However, the healthcare professionals felt that the length of the patient decision aid, and elements of its content may be a barrier to its use. Subsequently, the prototype was reduced to 40 pages. During *beta* testing of the shortened version in routine care, women who received the resource described its positive impact on their ability to make fertility preservation decisions and support them at a stressful time. However, practical difficulties emerged which impacted upon its wider dissemination in clinical practice and limited some elements of the evaluation planned.

**Discussion:**

Women receiving the decision aid within the cancer treatment pathway found it helped them engage with decisions about fertility preservation, and make better informed, values-based care plans with oncology and fertility teams. More work is needed to address access and implementation of this resource as part of routine oncology care pathways.

## Introduction

Worldwide, cancer patient survival rates are rising, resulting in an increased focus on helping people adjust to life after cancer treatment (1). For women of fertile years, one of the most distressing outcomes of some cancer treatments is its impact on fertility, potentially denying them the opportunity to have their own biological child (2, 3).

The impact of cancer treatment upon fertility may vary because of age, and how treatments inconsistently affect gonadal and uterine function. A Scottish population-based analysis showed a 38% reduction in the likelihood of a pregnancy rate after cancer treatments in girls and women aged <40 years across all diagnoses compared with the general population (4). It is recommended that fertility preservation (FP) treatments for women (such as egg, embryo, and ovarian tissue cryopreservation) should be discussed before cancer treatment starts, enabling women to consider their options, supported by the provision of written information/resources where possible (5–9). However, evidence suggests that many women are either not considered, or referred, for FP, are inappropriately referred (10, 11), or are poorly supported in making these complex decisions.

Patient decision aids are evidence-based resources supporting patients to make informed, values-based decisions between treatments (12, 13). Patient decision aids present information about the condition under focus and should provide the reader with unbiased information regarding their options/treatments and the associated benefits and risks (e.g., side effects) in a neutral way (14). They are intended to support but not replace good quality patient-doctor communication (15). A systematic review of published studies which had evaluated a patient decision aid in a randomised controlled trial compared to usual care (i.e., no intervention, usual care, placebo interventions, guidelines or a combination of these) concluded there was moderate to high quality evidence that patient decision aids enabled patients to be significantly more active in their treatment decision-making and become more knowledgeable, informed, and clear about their values compared to usual care with less subsequent decisional regret (15). Similar findings were also observed in a review of cancer treatment and screening specific patient decision aids which had also been evaluated in the context of a randomised controlled trial. The authors found that patients exposed to the patient decision aid had higher average knowledge scores, accurate risk perceptions and were more likely to be active in their decision-making compared to those exposed to usual care (16).

This paper describes the development and evaluation of a novel patient decision aid to support women of reproductive age at risk of losing their fertility because of cancer treatment to make FP treatment decisions entitled ‘Cancer, Fertility and Me’ (CFM).

## Materials and Methods

The current study used a prospective mixed-method observational study design and is reported following the Standards for UNiversal reporting of Decision Aid Evaluations (SUNDAE) guidelines (17).

CFM was initially designed in a print-based booklet form. It was developed and evaluated in three stages in line with best practice methodological recommendations from the International Patient Decision Aid Standards (IPDAS) (18), and consideration of other best practice decision science guidance (19, 20), past decision aid development and evaluation studies (21) and frameworks for assessing complex interventions (22). The three stages included the development of CFM with key stakeholders (stage 1), alpha testing the first prototype using past patients and healthcare professionals (HCPs) (stage 2) and beta (field) testing with patients and HCPs as part of routine clinical practice (stage 3). A protocol outlining these stages is described in-depth elsewhere (23) but summarised in **Supplementary Figure 1** and reported briefly below to meet the SUNDAE standards of reporting required and to highlight the places where the protocol changed from the initial version.

### Stage 1: Development of CFM – Framework, Theory and Process

This stage identified the theoretical framework to guide development of CFM and the ‘active’ components the resource needed.

#### Framework and Theory

The decision theory-centred, Ottawa Decision Support Framework (ODSF) (24, 25) was chosen as it is particularly suitable when the decision in question is preference sensitive and it provides systematic methods to identify the needs of different stakeholders when developing patient resources.

#### Our Prior Research Identified Women’s Needs

The scope, purpose and target audience of CFM was defined following a consideration of the evidence gathered from i) a systematic review of women’s values, treatment preferences and decision-making experiences (26), a longitudinal, mixed-methods study which explored the needs and experiences of women with cancer as they contemplated FP treatment decisions (the PreFer Study) (27) a mixed-method evaluation of a local FP service (28). In 2005, Thewes et al. (29) reported on the key fertility-related questions that needed answering from the perspective of young women with early-stage breast cancer – the largest cancer group facing a fertility preservation discussion which were also considered.

The results of the systematic review highlighted that FP decisions are preference-sensitive, time-sensitive, and typically stressful. They often occur around the time of cancer diagnosis, with FP interventions enacted before cancer treatment is initiated. External and internal factors can affect FP decision-making for women around cancer diagnosis in oncology services (26). External factors included barriers outside of the patient’s control (e.g., lack of/poor information provision, lack of knowledge and referral to fertility services amongst others). Internal factors highlighted the role of individual differences and subjective emotions as a barrier to FP decision-making including the fear associated with delaying cancer treatment, fear of aggravating a hormone positive cancer, and the fear associated with the consequences of a future pregnancy. These emotions can cause conflict for the patient about whether cancer or FP treatment should be prioritised (27), something that has been identified as a key factor in other areas of cancer-related decision-making (30).

The resource aims to better support women at risk of losing their fertility because of cancer treatment, to make the most appropriate FP treatment decision for them. This evidence suggested that a resource, administered around the time of cancer diagnosis in oncology services was most needed and desired. At the time the review was undertaken, it identified only two FP patient decision aids for women of reproductive age, both designed for women with breast cancer specifically (31, 32). It therefore highlighted how women with other cancer diagnoses (e.g., lymphoma) did not have a resource available to them to better support them with the FP treatment decision and that CFM should be relevant to women with cancer diagnoses beyond breast cancer.

To confirm the need, format and distribution plan, a PPI focus group (consisting of three women and a partner who had either faced the fertility preservation decision or missed out on such an opportunity) was also undertaken by GJ and JH to further confirm our CFM development plans.

#### Process

To guide CFM decision aid development, a multi-disciplinary group (n=24) comprising key stakeholders was convened, growing over time to consist of senior clinicians including paediatric, teenage and adult oncologists (n=6), a haematologist (n=1), fertility specialists (n= 4), oncology nurses (n=2), health psychologists/decision scientists (n=4), health service researcher (n=2), representatives from relevant charity organisations (n=3), and patient representatives (n=2). To further inform the content of CFM, we reviewed clinical guidelines on fertility preservation and cancer in females (5, 6), undertook an environmental scan to appraise the quality of existing fertility preservation resources (33) and undertook informal observations of local service delivery across sites in Yorkshire, UK undertaken as part of study set-up.

The key stakeholder group considered all the information referenced previously to identify key components to guide the design and content of a draft (e.g., *via* the discussion of potential CFM content during face-to-face meetings, and *via* email) until a consensus was reached. A design team (Making Sense Ltd) developed the illustrations and design of the resource.

### Stage 2: Face Validity (Alpha) Testing

Women with experience of FP decision-making in the context of a cancer diagnosis, HCPs and key stakeholders completed the Preparation for Decision-making Questionnaire (34), four items taken from the QQ-10 (designed to measure the face validity of a questionnaire) (35), and three open-ended questions relating to the acceptability and utility of CFM. Questionnaire data were analysed using SPSS Software (Version 24). Interviews were anonymised during transcription, uploaded onto NVivo and analysed using thematic analysis (36). An initial coding framework based on two interviews, was developed by four researchers (KSV, FD, NM and GJ) and was extended as appropriate. One researcher coded all interviews (KSV) and a second researcher (DM) coded two randomly selected interviews to check for differences. The generated themes were discussed between KSV, FD and GJ, until consensus was reached.

### Stage 3: Evaluation (Beta) Testing

In stage 3 we aimed to field test CFM as part of routine practice with 78 new patients recruited from oncology clinics and oncology HCPs (23). We had planned to use the referral model of recruitment, whereby women would be informed and offered the opportunity to take part in the study by their oncologist around cancer diagnosis. However, due to problems encountered with recruitment of women in oncology services, patient recruitment was further widened and extended from this initial protocol (23) (**Table 1**) to recruit women with cancer who were contemplating FP and had been referred to fertility services as well. Patients were recruited before starting cancer treatment (baseline). Baseline measures completed included the EQ-5D three level version (EQ-5D-3L) (37) and the traditional version of the Decisional Conflict Scale (DCS) (38). After women had completed at least their first round of chemotherapy (time 2) both patients and HCPs were asked to complete the EQ-5D-3L and the Decisional Regret Scale (DRS) (39) and invited to take part in a qualitative interview to explore their experiences of using CFM (time 2b).

**Table 1:** Changes made to the methodology used during the beta testing of the CFM patient decision aid.

	Planned	Actual
**Sample**	Women of child-bearing age (16 years +) with a new diagnosis of cancer	Women of child-bearing age (16 years +) with a new diagnosis of cancer
**Baseline**	**Recruited around the time of cancer diagnosis in oncology services**	**Recruited around the time of cancer diagnosis OR oncology or fertility services**
	Women who are interested in taking part will be given a study pack (containing CFM) by the researcher. They were asked to complete a demographic questionnaire, EQ-5D, Stage of Decision Making, Decisional Conflict Scale and STAI-6 before they can look at and read CFM.	Women who are interested in taking part were given CFM by the researcher. They were also asked to complete a demographic questionnaire, EQ-5D, Decisional Conflict Scale
**Time 1**	**Before the consultation with the fertility team member or patient’s next oncology appointment.**	**Before the consultation with the fertility team member or patient’s next oncology appointment.**
	Women will be given the following questionnaires to complete: the STAI-6, the Stage of Decision Making and the Preparation for Decision Making scale.	Removed
**Time 2**	**After the first round of chemotherapy has finished.**	**After the first round of chemotherapy has finished.**
**Time 2a**	Women will be posted the following questionnaires to complete: the STAI-6, the Stage of Decision Making and the Decisional Conflict Scale.	Removed
**Time 2b**	Qualitative interviews with patients, also asked to complete these questionnaires: EQ-5D and the Decision Regret Scale. Qualitative interviews with HCPs, who were also asked to complete a demographic questionnaire.	Qualitative interviews with patients, also asked to complete these questionnaires: EQ-5D and the Decision Regret Scale. Qualitative interviews with HCPs, who were also asked to complete a demographic questionnaire.

The same analysis methods were applied as in Stage 2, with the exception that a lay advocate (JG) independently coded three interview transcripts. We used the criteria from the SUNDAE Checklist (17) seeking to understand: i) How much and which components were used, ii) the degree to which it was delivered and used as intended (“fidelity”), and iii) any anticipated and unanticipated consequences. Count data was gathered to measure the number of CFM patient decision aids given to women and HCPs, website views, and the downloads of the PDF version.

A subset of women were also recruited in an online survey *via* social media to a closed Breast Cancer Now Facebook group (formerly Breast Cancer Care) and the support group Cancer and Fertility UK to give feedback on the CFM patient decision aid.

## Results

### Stage 1: Development of CFM

The development of CFM was an iterative process, with over 100 versions created before the first 60-page prototype (version 1.0) was produced.

**Figure 1:**
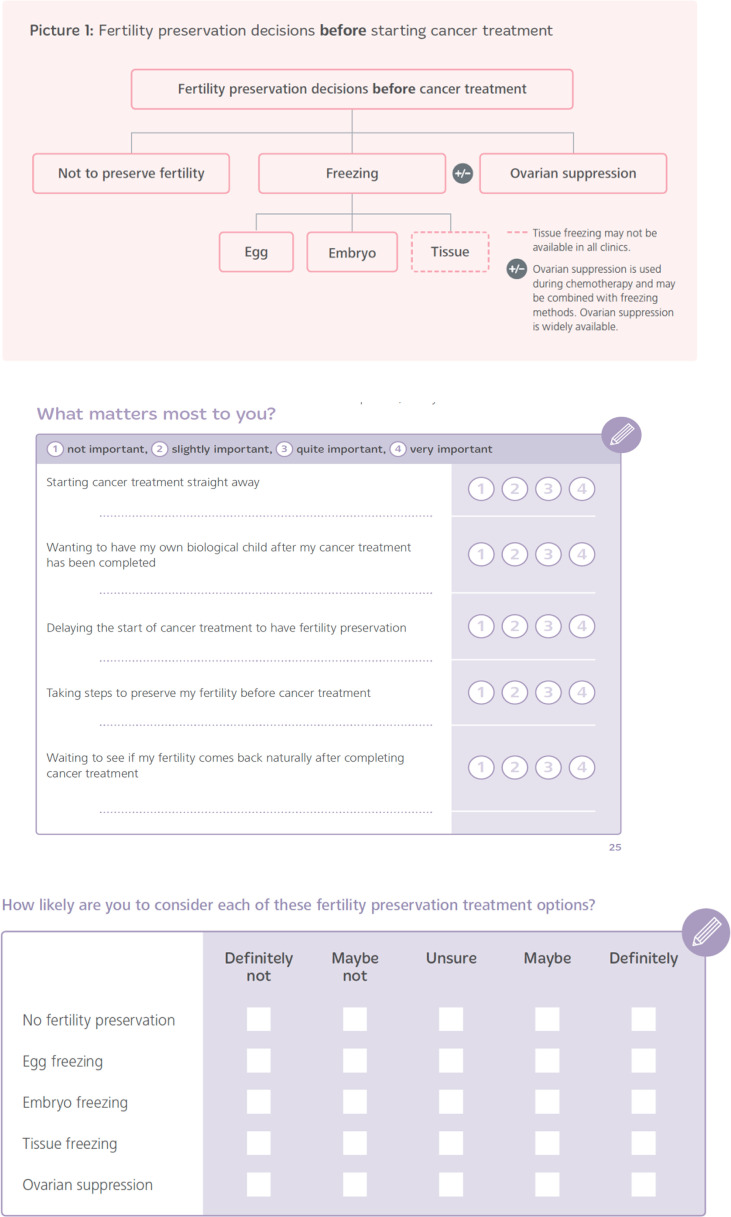
Examples of a decision picture and the values clarification exercises in CFM. ©

The key components in the patient decision aid include:

* Explicit information about, and description of, the decision to be made (i.e., helping women with cancer to make decisions about FP treatment before starting cancer therapy),
* Describing the health problems (i.e., cancer, fertility and the reproductive system, potential impact of cancer treatments on fertility),
* Providing information, in visual, text, numerical (%) and table formats, to describe treatment options (including benefits/harm/consequences), which also included avoiding or postponing treatment (i.e., no FP, egg, embryo or ovarian tissue freezing, with or without ovarian suppression),
* Tailoring this information for each of the following factors (e.g., relevant patient group, features of the intervention - including where the FP treatment option may be considered a newer treatment method, implications for achieving a successful pregnancy, and chances of cancer recurrence),
* Providing guidance for communication and deliberation about the FP decision with HCPs and important others (e.g., *via* suggested questions to use, spaces to write and list what they like and dislike about each option, exercises to think about the importance of referral, and help the women clarify their own values) (**Figure 1**),
* Information on the other fertility decisions to consider during and after cancer treatment, represented by a decision map (30) to each stage of the cancer pathway before, during and after cancer treatment,
* Other components (i.e., information about useful contacts, sources evidence, glossary, the team),
* A one-page summary table, for potential use as an option grid. Columns are the fertility preservation options and the rows the frequently asked questions.

To support health literacy, reading levels were obtained for each page. The resource had an overall Flesch-Kincaid reading score of 52.14, which is at an average level to easily be understood by 15- to 16-year-olds.

### Stage 2: Face (Alpha) Validity Testing (Version 1.0, Dated August 2016)

Eleven women, 10 HCPs and four other key stakeholders from Yorkshire Cancer Research, and the British Fertility Society, were recruited to the alpha testing of CFM (**Table 2**). From the 10 HCPs in clinical roles, there were five medical consultants and one junior doctor, three nurses and a social worker (mean age = 45.2 years old, range = 30-58 years). Their clinical specialities included breast cancer, teenage and young adult oncology, reproductive medicine, late effects, paediatric oncology, haematology and bone marrow transplantation. QQ-10 questionnaires showed both patients (mean = 4.3, range = 3.3 – 4.7), and HCPs (mean = 4.2, range = 2.55 – 4.91) found the resource acceptable (**Table 3**). The resource also appeared to be considered useful in preparing women for FP treatment decisions (mean = 4.3, range = 3.9 – 4.5) and HCPs (mean = 4.2, range = 4.0 – 4.6). When converted out of 100, this resulted in a score of 82.3 for the women and 81.0 for the HCPs (**Table 3**).

**Table 2:** Demographic and clinical characteristics of the patient and HCPs in the alpha and beta testing stages of the study.

Category	Alpha Testing Patients (n=11)	Beta Testing Patients (n=41)
**Mean Age (range)**	33.0 (22-44)	32.12 (16-43)
**Type of Cancer, N **		
Breast	8	27
Lymphoma		5
Cervical		2
Brain		2
Aplastic Anaemia	1	2
Bowel		1
Germ Cell (Ovarian)		1
Rectal		1
Ovarian		1
Osteosarcoma		1
Head and neck	1	
	1	
**Ethnicity N (%)**		
White British	9	28 (68.5%)
White Other*		6 (14.6%)
Pakistani		4 (9.8%)
Chinese		1 (2.4%)
Black Caribbean	1	1 (2.4%)
Indian		1 (2.4%)
Hispanic	1	
**Relationship Status, N **		
Married	4	19
Living with partner	2	12
Single	5	8
Separated		8
Prefer not to say		8
**Education, N**		
O-Level/GCSE		7
A-Level/GCE	3	6
HND/Diploma	1	5
Degree	5	13
Higher Degree	2	6
Missing / None		1 / 3
**Number of Children, N**		
One	7	24
Two	2	8
Three	2	5
Five +		2
Not reported/Missing		1
		1

**Table 3:** Results of the QQ-10 scale and the preparation for decision-making scale during alpha testing.

PtDA alpha testing with patients: results of the QQ-10 scale			
	Results: Mean (SD)		
**Was the booklet** **(Scoring: 1 = strongly agree; -4 = strongly disagree)**	**Overall** (n=22)	**Patient & Service users** (n=11)	**HCP & Key stakeholders** (n=11)
**Too long**	2.9 (1.5)	3.3 (1.3)	2.55 (1.3)
**Embarrassing**	4.8 (.5)	4.7 (.7)	4.91 (.3)
**Upsetting**	4.4 (1.3)	4.3 (1.4)	4.55 (1.2)
**Complicated**	3.9 (1.2)	4.4 (.7)	3.45 (1.5)
**PtDA alpha testing with patients: results of the preparation for decision-making scale**			
**Did the booklet…** **(Scoring: 1 = strongly disagree; -5 = strongly agree)**	**Results: Mean (SD)**		
	**Overall** (n=22)	**Patient & Service users** (n=11)	**HCP & Key stakeholders** (n=11)
**Help you recognise that a decision needs to be made**	4.3 (1.0)	4.36 (.7)	4.2 (1.2)
**Prepare you to make a better decision**	4.3 (1.1)	4.3 (1.2)	4.3 (1.0)
**Help you think about the pros and cons of each option**	4.3 (1.1)	4.3 (1.3)	4.3 (1.0)
**Help you think about the pros and cons are important**	4.2 (1.0)	4.3 (1.2)	4.1 (.9)
**Help you to know that the decision depends on what matters most to you**	4.3 (.7)	4.5 (.5)	4.1 (.9)
**Help you organise your thoughts about the decision**	4.3 (.8)	4.3 (.7)	4.3 (1.0)
**Help you think about how involved you want to be in this decision**	4.0 (1.2)	3.9 (1.4)	4.0 (0.9)
**Help you identify the question you want to ask your doctor**	4.5 (.9)	4.3 (1.2)	4.6 (.5)
**Prepare you to talk to your doctor about what matters most to you**	4.3 (.9)	4.4 (1.2)	4.3 (1.0)
**Prepare you for a follow-up visit with your doctor**	4.2 (1.0)	4.3 (1.2)	4.2 (.9)
**Summed score (Mean)**	4.3 (.9)	4.3 (.9)	4.2 (.9)
**Preparation for Decision-Making Scale (Score 1-100)**	81.7	82.3	81.0

Thematic analysis generated three key themes from the patient and HCP interview data. These related to resource design and content, its use in supporting FP decision-making and, its use in practice. The feedback on the resource design and content including the colours, paper, font size, general layout, order of content, and infographics were very positive. However, the length of the resource was raised as a concern by some HCPs. All patients and most HCPs also felt the resource supported decision-making, although some of the text was considered too complex by a few HCPs, and therefore they questioned whether CFM would be helpful in supporting patients to make FP decisions. With regards to the usability of the resource, most HCPs felt that the length, its complex content and the need to maximise survival over fertility would be a barrier to use at the time of a stressful cancer diagnosis. In contrast, only four women expressed concerns about complexity or length.

The practical recommendations for changes that were raised by patients and HCPs are shown in **Tables 4**, **5** respectively. Following a discussion and consideration of these findings with the steering group, the CFM decision aid underwent a set of revisions and amendments before being used in stage 3. These revisions shortened the resource by 20 pages.

**Table 4:** Practical recommendations made by patients following alpha testing.

	Recommendation	Checklist ✓ Changes made × Changes not made	Reason
**Add**	Add room for other factors that may influence decision, e.g. social factors (STH10)	×	Not specified enough, covered some social aspects already
	P.34 add “yes no maybe” (STH10)	×	No, but we did change the response
**Change**	What to do if patients disagree with doctors’ recommendations (LTH8)	×	Unethical, decision-aid is supposed to be neutral
	Consent form examples, emphasise quantity (LTH8)	×	Can differ between hospitals, leading?
	Surrogacy versus carrying own baby (LTH8)	×	Not within remit
	What happens at the clinic (LTH9)	×	Felt a lot of information already, but we did update the text (e.g. changes from fertility expert to fertility care team)
	Signpost HEFA website (LTH9)	×	Already done
	Case studies, possible side effects (STH10)	×	Would bias the decision aid, we do mention side-effects
	How relatives can support decision maker	×	Already included
	Possibility of fostering, does having had cancer affect chances? (SU3)	×	Felt inappropriate
	Adoption issues after CT (SU3)	×	Felt inappropriate
	Add POSITIVE cancer trial results (SU4)	×	
	P12, graph: add shaded area (STH10)	✓	
	Move p. 35 to the end (SU4)	✓	
**Omit**	Can be challenging numbering 1-4 (STH9)	×	
	Page 10, cut where it says “use space” as the women could bring a notebook (STH10)	✓	

**Table 5:** Practical recommendations made by the healthcare professionals and key stakeholders following alpha testing.

	Recommendations	Checklist ✓ Changes made × Changes not made	Reasons
**Add**	Ovarian suppression can be used irrespective of egg or embryo freezing (LTH1)	✓	
	Ovarian transposition as an option (KS2)	✓	But not in the graph
	Add sections about how partner feels about options (STH1)	×	
	Dotted line to say not all treatments are available in all clinics (STH4)	×	
	Question: “I am getting symptoms of menopause post CT, where can I go for help?” (STH4)		Already in
	Need a separate small leaflet to say that things can affect your fertility but unfortunately in your case we can’t do anything to preserve for these reasons (LTH2)	✓	But not as separate leaflet
**Change**	Despite already simplified, might still be too much for ‘average’ patient (STH4)		Tried modifying, Flesch reading age
	Better to not give success vs failure rates due to differences in clinics (LTH1)	×	
	Consider revising statistic that menopause in cancer survivors can occur 5-10 years early; p.12 (KS3)	✓	
	Ovarian suppression not new (KS2)	×	We already said ‘newer’ not ‘new’
	Text: Consistency, i.e. side-effects or side effects, throughout the booklet (KS4)	✓	
	Avoid italics as it makes it harder to read (KS4)	✓	
	Some corrections regarding grammar and spelling (KS4)	✓	
	Don’t’ make it shiny paper, so that patients can write on it (STH4)	✓	
	Language issue: ‘loss of fertility’ as too dramatic? (STH3); subfertility possibly? (LTH3)	×	Too clinical
	Use ‘eggs’ not ‘follicles’ (LTH2, STH5)	✓	
	Graph 2, page 12, needs correcting (LTH3)	✓	Yes to cancer treatment, edited this graph significantly
	Change the axes on Table 1 (LTH5)	✓	
	Link p.24 with purple section (STH7)	×	Unclear
	Make the graphs and tables less academic (STH7)	✓	Yes, e.g. replaced chemotherapy/radiation with cancer treatment, modified language
	Some graphs repetitive, e.g. page 11 (STH7, STH1)	×	Not correct
	Don’t put ovarian suppression in dotted line (STH4)	✓	
	Questions for reflections too theoretical? (LTH3	✓	Did cut down number of questions
	Less space for questions (STH4)	✓	
	Add: “Questions to discuss with other people you are close with, not just partner” (STH5)	✓	
	Abridged version for some? (LTH2)	×	
	Too long, too complex, too long-winded (LTH3; STH7)	×	We did cut down from 60 pages to 40
	Break booklet up in individual sections, give out what’s appropriate? (LTH5)	×	
	Emphasise that it’s a workbook, not another bunch of leaflets	✓	
**Omit**	All cancer background (LTH3)	×	
	Unsure if summary tables add value (LTH6)	×	Investigated in Evaluation Phase
	Diagram p12 too technical, take out (STH3	✓	Modified this slightly
	P.17: make outline stand out, bold (KS4)	✓	
	Grey boxes: ensure they are within the same margins, on same part of the pages (KS4)	✓	
	Decision pictures: Could omit, but best keep (STH1)	×	
	Flowcharts possibly redundant, as text says the same (LTH1)	×	
	Could out some space potentially (STH3)	✓	Yes, reduced overall length
	Cut questions, add extra sheet (LTH3)	✓	Cut some questions, no extra sheet
	Cut down different treatment information (LTH5)	×	
	Get rid of option table (STH5)	×	

### Stage 3: Evaluation (Beta) Testing (Version 2.0, Dated February 2017)

Forty-one women were recruited (13 from oncology clinics and 26 from fertility services) (**Table 2**).

In consideration of FP options at baseline and before using CFM, 17 women (41%) felt they were considering their options right now, with 12.8% who had not yet begun to think about their options. Thirty-seven (90.2%), completed all 16 items of the decisional conflict scale at baseline (**Supplementary Figure 2A**). The total mean score was 30.7 (SD: 21.4; range: 0 - 76.6 on a scale of 0-100) indicating lower than average levels of decisional conflict, although the uncertainty sub-scale exceeded the threshold of ‘higher’ decisional conflict. Baseline EQ-5D-3L mean scores revealed low levels of problems in the five areas of quality of life. With the exception of usual activities (*p* = 0.018), there were no other significant differences in quality-of-life scores based upon the EQ-5D data pre and post receipt of CFM.

Thirty-one women and three HCPs were subsequently interviewed, with 10 women declining to take part at this stage primarily being too ill to be interviewed. Recruitment/administration of the resource in the beta stage appeared to fall to the same small number of HCPs across the two centres. Due to work commitments of the staff, we were only able to approach three - all of whom accepted. Prior to completing the interview, 29 women (70.7%) completed the decisional regret scale (DRS). The mean DRS score was 42.6 (SD = 7.7; range = 25-65 of a scale of 0-100) (**Supplementary Figure 2B**). There were no significant differences in total DCS scores between those women who subsequently took part in the qualitative interview and those who did not.

### How Much of CFM Was Used?

Overall, CFM was highly regarded and the majority of the components of CFM were used (**Table 6**). Women highly regarded the colours, graphs, statistics presented, tables and flow charts, expressing that they were clear and focused and provided a helpful summary of the information in order to assist in decision making:

**Table 6:** Which components of the CFM resource were used?

Sundae Checklist – Point 21 (part a) Theme components used:	Patient Quote	Age	Cancer type	Recruited in oncology or fertility?
Graphs and tables	I thought they [graphs] were really clear, really helpful.	38	Breast	Oncology
Glossary	No, I think it’s a good way to – if somebody says something and ‘oh, I don’t quite understand what that is’ and then you can have a quick look.	36	Breast	Fertility
Decision pictures/flow charts	I did, and again – a lot more beneficial and helpful sometimes than what the actual information content could be.	34	Breast	Oncology
Decision pictures/flow charts	And again I think they’re hugely beneficial because it is that scrambled brain.	34	Breast	Oncology
Decision aid section	I did, I didn’t put the ticks, I didn’t give it a score but we sort of…. I mean it says do it before your treatment starts and I did do it before the treatment started…	37	Breast	Oncology
Decision aid section	… I just thought how I felt with each statement and it came to the conclusion that you know, I didn’t want to do any of them … it affirmed what I wanted to do…	20	Lymphoma	Fertility
Graphs and tables	I think the thing that I remember the most was this diagram on Page 7 [graph 1] and for all the people that are putting off having kids…	43	Breast	Oncology
Sections used	So the green bit [Introduction] because it’s describing what the booklet’s about. Then the blue bit [Other fertility decisions to consider] – it looks like a biology text book.	30	Breast	Fertility
Sections used	I used the blue bit [Other fertility decisions to consider].	30	Breast	Fertility
Sections helpful	Egg freezing and embryo freezing.	38	Breast	Oncology
Sections helpful	I like this graph and picture and those tables … Pink section [Options] and this [Graphs 1 and 2].	29	Breast	Fertility
Sections helpful	I think the section for ovarian suppression, it helped to make a decision to do something…	40	Breast	Fertility
Sections helpful	It was just on the benefits of the ovarian suppression and things like that…	38	Breast	Fertility
Sections helpful	I just kept going over the sections where it gives you the options and I just kept reading the statistics. I thought having the statistics was really helpful.	25	Ovarian	Oncology
Sections helpful	Just the back table [affected the decision].	30	Breast	Fertility
Sections unhelpful	[How cancer treatment affects your fertility] made me even more depressed than I already was.	25	Ovarian	Oncology
Sections not used	Again I didn’t use it [decision aid section]. I did go through it but I didn’t use it because of what I’d actually decided on and what I was thinking and what I was going through.	34	Breast	Oncology
Sections not used	I didn’t use it if I’m honest [signposting].	23	Germ cell - Ovarian	Oncology
Sections not used	Oh, I didn’t even notice these graphs!	25	Ovarian	Oncology
Not used at all	It was laziness to be honest. When I got sent the attachments I had quite a few appointments going on around then and that’s when I got taken into hospital as well. So after that I completely forgot.	21	Lymphoma	Fertility
Writing	I just kind of answered what it said so it takes my opinion on things and I write down what I like, what I liked about it, what I didn’t – literally how it says. Which I thought was quite a helpful way to organise my thoughts and balance up my opinions really…	20	Lymphoma	Fertility
Note taking	Yeh, cos you make like a note of what you want to ask a nurse or a doctor.	16	Lymphoma	Oncology
Realisation of age	My biggest eye opener was the graph. I remember looking at it and thinking right, my treatment is only here which means that my fertility would be technically lower anyway and it helped guide the decision a little bit that I made.	34	Breast	Oncology
Realisation of age	… That quite surprised me because I didn’t realise that, for example I’m in thirties so I didn’t realise I had so little chance! And I think Graph 2, when I look at it, it freaks me out actually….	29	Breast	Fertility
Understanding and knowledge	… We went down the embryo bit. Just because we felt it was the better option. So we did read that a little bit…	30	Lymphoma	Fertility
Understanding and knowledge	… Well just even having the sections about how cancer affects your fertility because it hadn’t been something I really considered when I first got the diagnosis so it was useful to understand that there would be an impact…	30	Lymphoma	Fertility
Understanding and knowledge	… I was more worried about the hormone therapy so it was this that helped me understand a bit more about how it might affect me.	36	Breast	Fertility
Understanding and knowledge	It was the explanation of each of them – the fertility options. They were what I’d have needed to know.	20	Lymphoma	Fertility

*“The colours I think is a really good choice. They’re kind of calming colours.” (Patient, age 20, lymphoma, fertility clinic)*

*“That [summary table] was really helpful. You go through all of the information and you’re desperately trying to absorb it all…’’ (Patient, age 38, breast cancer, oncology clinic)*

*“A lot of time and effort I think has been taken and that makes me feel better when I’m reading it because it makes me feel like it’s come from a source that actually cares and is reputable.” (Patient, age 29, breast cancer, oncology clinic)*

*“I actually liked the fact it was A4 cos everything else is A5. It somehow separated it out and made it not about cancer.” (Patient, age 40, breast cancer, oncology clinic)*

These components helped to facilitate realisation of information and evidence, as well as increase knowledge awareness:

*“I just kept going over the sections where it gives you the options and I just kept reading the statistics. I thought having the statistics was really helpful.” (Patient, age 40, breast cancer, oncology clinic)*

*“This is a lot better, I can read through it myself in my own time and it is not biased, it is telling me facts and options.” (Patient, age 29, lymphoma, fertility clinic)*

*“I think it strengthened my decision, it was something I might not have been clear on without the booklet.” (Patient, age 30, lymphoma, fertility clinic)*

*“It affirmed it. What it meant is I understood what I was doing. It meant that I understood the other options properly and I knew what I was turning down essentially.” (Patient, age 20 lymphoma, fertility clinic)*

However, not all women used all components of CFM, for example, some women stated that they did not always use the spaces provided in the booklet to make notes, or they did not complete the decision-making values exercise in the booklet. It appeared that the majority of women may not put pen to paper and would refer to these prompts when thinking through the process of decision-making. For the few women who did write in the booklet, they described the value of being able to take it to the consultation as an aide-memoire for discussion:

*‘‘I just kind of answered what it said so it takes my opinion on things which I thought was quite a helpful way to organise my thoughts and balance up my opinions really. It also makes like a note of what you want to ask a nurse or a doctor.’’ (Patient, age 20, lymphoma, fertility clinic)*

### Anticipated Consequences of Using CFM

**Table 7** shows the anticipated consequences of using CFM. No unanticipated consequences arose. For some, it helped to reduce feelings of denial, highlighting the reality of their situation and the decision faced. It also helped to convey information to friends and family, and facilitated conversations about cancer and fertility to take place with the support of others:

**Table 7:** Anticipated consequences of using CFM.

Anticipated Consequences
**FRIENDS AND FAMILY CONVEYED THE INFORMATION** ….but to be fair it was X [partner] that was looking and just breaking it down and telling me – cos I didn’t want to over think things because I’d got too much going on in my body that I didn’t want to be putting a load of other pressure on me. *(Patient, age 30, lymphoma, fertility clinic)* I didn’t want to see it [the word *cancer*]. So that’s why I kind of left it to him to filter for me. *(Patient, age 30, lymphoma, fertility clinic)* He [partner] probably understood it all a lot more than me……then he broke it down for me, explained to me in the way that he knew I would understand at the time……cos me ‘ead wasn’t in it. *(Patient, age 30, lymphoma, fertility clinic)* When I’d like read bits and kind of like I’d got down to between two and then sent pictures of the information to my parents and they gave me their opinions. *(Patient, age 23, ovarian cancer, oncology clinic)* Yeh, me mum helped me with making the decisions cos she’s been through it [fertility treatment]. She sat with me and we talked about it for a good two days straight. *(Patient, age 25, ovarian cancer, oncology clinic)* ….in the pink bit [treatment options] it was a bit too detailed for me….there was a little bit too much of what you had to go through. I’d like it more like just a summary and then if I wanted to know about one I’d probably want to go……I just found it a little bit upsetting. *(Patient, age 28, breast cancer, oncology clinic)*
**DENIAL** It’s really useful to have and you need to have it because if anything happened we need to read it. But it is good that it’s highlighted so I can just quickly go ‘I’ll skip through that bit’. *(Patient, age 29, brain cancer, fertility clinic)* So when you’ve got that [the title] staring at you, it’s ‘ard and I get upset. *(Patient, age 30, lymphoma, fertility clinic)* ….when I’d just been diagnosed, to have that [the word Cancer] on the front of it, that’s like just jumped out at you and I didn’t want to see anything like that. *(Patient, age 30, lymphoma, fertility clinic)* I’d got in my mind as long as I weren’t gonna die and as long as everything was fixable I didn’t like seeing it all black and white like. *(Patient, age 34, breast cancer, oncology clinic)* Cos you don’t want to see all these keep jumping out [flicks through booklet]……I asked him to hide it. All the paperwork he hid from me in the end cos I didn’t want to see it. *(Patient, age 30, lymphoma, fertility clinic)* The booklet….goes on to look at the percentage chance of you conceiving…….I sort of skipped over that. *(Patient, age 38, breast cancer, oncology clinic*) I know there’s the bit about how the cancer can affect it, how the treatments can affect it and that can be – I know for me cos I like to try and be positive and everything – it can be a bit of a, like it brings me down a little bit so I try and avoid sections like that. *(Patient, age 29, brain cancer, fertility clinic)* [How Cancer Treatment Affects Your Fertility] made me even more depressed than I already was. *(Patient, age 25, ovarian cancer, oncology clinic)* Some of it was overwhelming, some of it scared me. *(Patient, age 38, breast cancer, oncology clinic)*
**EDUCATING OTHERS** Her interest was because she’s a medical student and this is obviously not an area that she’s come across yet. So it was, all the ideas were new to her, which I’d heard of pretty much everything in here so it wasn’t such news to me. So it really was, she just takes the opportunity to learn something as she goes along. *(Patient, age 43, breast cancer, oncology clinic)* No. I mean she’s [patient’s mother] read more of the booklets and taken more of that, I think to get a bit more of an understanding whereas because it happened so quick and the actual treatment was so quick I think I was just in a whirlwind of getting from A to B whereas she took more information on than I did. I think it kind of went straight over my head most of the stuff cos it was just like in that moment what I had to do. *(Patient, age 28, breast cancer, fertility clinic)* He [partner] thought it was quite informative and it gave him some things to look up online. *(Patient, age 38, breast cancer, fertility clinic)*
**EMOTIONS EXPRESSED** ***RAISING EXPECTATIONS*** So I didn’t want to get completely like me ‘hopes up’ because I didn’t know that it was gonna get taken away. *(Patient, age 30, lymphoma, fertility clinic)* ***SELF BLAME*** I kept saying what am I doing to me body? Why am I doing this when I ‘ve got something else that I’m going to have inject into me body? *(Patient, age 30, lymphoma, fertility clinic)* ***GUILT*** It was as if I was checking facts and even after I’d made the decision, because I then sat in the fence a little bit and felt so guilty….yeh. *(Patient, age 34, breast cancer, oncology clinic)* ***SHOCK*** ….but I wouldn’t ever have thought that a person at sixteen had fifty per cent chance of getting pregnant but then at thirty years old they get ten per cent. I would never have known that. I mean that’s quite – that’s the bit that shocked me overall, out of the booklet. *(Patient, age 30, breast cancer, fertility clinic)* ….literally I were in shock that much there were bits that I didn’t….I don’t remember seeing all this bit ….I either didn’t read it properly at all cos of everything that was going on…… *(Patient, age 34, breast cancer, oncology clinic)*

*“… mum helped me with making the decisions cos she’s been through it [fertility treatment]. She sat with me, and we talked about it for a good two days straight.”*

*(Patient, age 25, ovarian cancer, oncology clinic)*

The process of sharing the information with friends and family also worked as a tool to help inform others:

*“she’s [patient’s mother] read more of the booklets……. whereas because it happened so quick, and the actual treatment was so quick I think I was just in a whirlwind of getting from A to B whereas she took more information on than I did.” (Patient, age 28, breast cancer, fertility clinic)*

Some women regarded the content of CFM upsetting – particularly how cancer and its associated treatment can affect fertility, the consequence being they chose to avoid reading those sections:

*“I know there’s the bit about how the cancer can affect it, how the treatments can affect it … it brings me down a little bit so I try and avoid sections like that.” (Patient, age 29, brain cancer, fertility clinic)*

Following the reading of CFM content guilt was expressed by some women who were contemplating undertaking FP at a time when their body was having to undergo challenging cancer treatment:

*“Why am I doing this when I’ve got something else that I’m going to have inject into me body? ”(Patient, age 30, Lymphoma, fertility clinic)*

### Degree to Which CFM Was Delivered and Used as Intended (“Fidelity”)

Our intention for recruitment was that all women of reproductive age at risk of losing their fertility because of cancer treatment, would be offered the opportunity to take part in the study and read the resource around the time of their cancer diagnosis in oncology.

In practice, it was the fertility clinics which became the primary source of delivery. Operational pressures, significant clinical workloads (as a result of staff shortages) and competing clinical priorities (urgency of the cancer treatment) at a particularly stressful point in the pathway for patients (i.e., diagnosis) particularly within the cancer setting, perhaps meant the study could not always be prioritised:

*“You’ve got a short window of opportunity to take this opportunity and in that sense there’s pressure, there’s a lot of pressure. It’s a pressured decision-making process………. we have 650 new cancers a year and 300 women living with secondary breast cancer and there’s seven of us and we’re never all here.” (HCP, oncology clinic)*

*“The more there is to read when people are in a state of distress, I think the harder it is to concentrate and focus.” (HCP, oncology clinic)*

*“I did not [read CFM]. If I would have got the information given to me in hospital by [oncologist], definitely I would read it.” (Patient, age 36, breast cancer, fertility clinic)*

However, some HCPs expressed that the low recruitment rate was simply because:

*“The majority of women that I gave it to have a clear vision in their mind as to what they wanted.” (HCP, oncology clinic)*

This was echoed in some women’s responses to us:

*“The doctor that told me I’d got cancer, he must have spoken to me for a good hour and I literally couldn’t tell you a single thing he said to me. It’s like white noise, you just don’t hear anything.” (Patient, age 29, breast cancer, oncology clinic)*

*“When in the midst of being told that I’m about to have chemo I was given it and told that it’s to help make my decision on fertility.” (Patient, age 23, ovarian cancer, oncology clinic)*

*“I think I already knew before I read the book.” (Patient, age 29, bowel cancer, fertility clinic)*

Thus, the delivery of the resource, particularly in the cancer setting, was often not as had been intended with the responsibility often falling to the oncology nurses instead. There was limited (or no) chance for women to sit and discuss the contents of the resource with patients describing that their HCP did not take the opportunity to signpost their patients to useful information that would help them to consider FP decisions,

*“I do wonder if there was a part my age played in that a little bit, potentially, I’m not sure. I think if maybe I was under thirty, in that twenty to thirties bracket, there may have been more of a ‘you really ought to be taking a while to think about this … I think, somebody younger, there may have been more of a push.” (Patient, age 34, breast cancer, oncology clinic).”*

*“So the booklet was really helpful…. because the consultant himself was very much just trying to push me to just not even think about that, but we wanted to think about that – being referred to the fertility clinic, and the booklet did help us know we’re making the right choice.”* (*Patient, age 38, breast cancer, fertility clinic)*

*“I weren’t given any option by my oncologist, so he never went through any of this with me. So obviously that booklet [CFM] was very informative.” (Patient, age 23, cervical cancer, oncology clinic)*

For some women this meant that they were not aware of the information in CFM, until it was too late in their cancer treatment process. Women often turned to the resource in retrospect with the realisation of how crucial it could have been to them when making their FP decisions:

*“I wish I’d read it beforehand so I would have actually known what to ask him [the oncologist] you know? Cos you don’t actually realise what questions you want to ask at that time. Cos obviously everything was just done so quickly, and you can’t think at the time of what you need to do, what you shouldn’t do and stuff like that.” (Patient, age 34, breast cancer, fertility clinic)*

### Facebook Survey

Finally, of the 29 participants who consented to take part in the social media study, 10 completed the online survey. Women were diagnosed with breast cancer (n=4), bowel cancer (n=2), childhood pelvic rhabdomyosarcoma (n=1), lymphoma (n=1) and cancer of the uterus (n=1) (missing n=1). The mean age was 36.8 (SD = 5.29; range = 28 – 43). Five were currently undergoing cancer treatment. The responses further confirmed the acceptability and value of CFM as a resource to support the FP decisional needs of this patient group:

*“I think this is brilliant and much needed. I feel it’s essential ALL girls/women with cancer are aware of ALL options prior to starting treatment.” (Participant, age 41*, *childhood pelvic rhabdomyosarcoma, Facebook survey)*

*“I think it is really important for women to have access to this information. The thought of losing my fertility was the most upsetting part of my cancer experience.” (Participant, age 31, breast cancer, Facebook survey).*

## Discussion

The aim of this study was to develop, and field test a novel patient decision aid to support women, aged 16 years and older, diagnosed with *any* cancer, to make FP treatment decisions before the start of their cancer treatment. It was well received during both the alpha and beta testing stages, with women describing how it helped them engage with decisions about FP, and make better informed, values-based care plans with their oncology and fertility care team.

Of the 41 women taking part in the beta testing study, only a quarter felt they had already made their decision and were unlikely to change their mind. With the majority still uncertain, the need for using such a resource very early in the cancer pathway is thus indicated. Low levels of fertility-related knowledge have been linked to increased decisional conflict in young patients with breast cancer (40) and a perceived lack of overall support for women (41).

Whilst the quality of the tool was acknowledged, there were difficulties in wider dissemination in clinical practice thus limiting extensive evaluation, such that CFM was not delivered in the same way for all participants. This concerns the ‘fidelity’ of a decision aids use and implementation (17). Our intention was that the tool would be administered in cancer services around the time of diagnosis. Instead, most women were recruited in fertility services. Discussions with cancer HCPs provided several explanations for the low recruitment figures, including the priority to decide and deliver the cancer treatment, demanding workloads, the need to protect women from further stress and the perception or assumption that many women had already made their decision beforehand. It is undoubted that some patients may have been unsuitable to partake in this research study. However, several women who missed recruitment to the study in cancer services then welcomed the opportunity to take part in the study once approached in fertility services.

It can be very difficult for oncology healthcare professionals to have fertility discussions in the context of a recent cancer diagnosis (42). It depends upon members of the oncology team using their best judgements to communicate information related to prognosis and treatment options which are complex and frightening (43). They have also described lacking the knowledge to advise appropriately during their fertility discussions with women and have requested more specialised resources to support them during these consultations. For example, one study highlighted that 87% of oncologists expressed a need for more specialist FP information, and that only 38% of oncologists routinely provided patients with written information (44). Similarly, a survey of 273 physicians involved in the care of breast cancer patients was conducted to explore fertility and pregnancy issues (pre and post cancer diagnosis) in young women with breast cancer. Between 17.6% and 48.4% reported having inadequate knowledge about the FP treatment options and it was concluded that further educational initiatives are needed in the future to better inform and support these physicians (45). In our study, one healthcare professional we interviewed described how lack of data on the ways that socio-demographic factors such as body mass index affected FP treatment outcomes made it difficult to know what best to do clinically in certain situations.

Whilst we attempted to prepare the clinical staff at several points throughout the study by targeting communication skills training on how to incorporate the utility of CFM in their doctor-patient communication/relationships (e.g., by providing scripts) more needs to be done to prepare, support and upskill staff in this new area of practice, especially regarding the ‘referral model’ of recruitment (46) and use of CFM in routine practice as a tool to support shared-decision-making. In relation to recruitment and administration of CFM, we found this was often delegated to the oncology nurses (particularly the breast cancer nurses). Therefore, one possible solution to addressing low recruitment rates in these types of studies may be to design patient recruitment around oncology nurses rather than the oncologists/surgeons. Implementation of FP patient decision aids in routine clinical care may also be improved if delivered by nurses. However, one possible explanation for why the resource was not given out as intended was because of a possible lack of clarity from within the multidisciplinary clinical team regarding whose role it was to have the FP consultation with patients and at what time point in the patients care pathway. All HCPs, including nursing staff will only be able to undertake this work, if it becomes clearly defined within their clinical roles, responsibilities and expectations. For this reason, we are currently in the process of developing a more comprehensive training package that may better support HCPs (including nurses) working across cancer services to use CFM in consultations. We also plan to undertake future work to identify and evaluate where in the clinical pathway the resource may work best; the findings also suggest that CFM may work better if it is administered to women in advance of seeing the oncologist. The use of the one-page summary table as an option grid on its own also needs evaluating.

Planned analyses were not possible with a small sample size and as such our focus shifted more to interpreting the qualitative interview data. As such interrogating quantitatively whether patient demographic characteristics which are known to influence FP treatment decision-making such as relationship status, age and parity (26) was not possible. We also did not achieve the diversity in the sample we had hoped for in terms of ethnicity for example to explore these issues. Despite this, from the qualitative interview data overall, it appeared that very similar experiences of FP decision-making arose, regardless of personal characteristics. With the exception that some women considered that their age and financial situation had influenced the FP treatment options discussed with them, and the decisions they made. Furthermore, those with dyslexia, poor eyesight or who did not speak English as a first language described how reading CFM was affected by these personal characteristics. This requires further detailed analysis and future studies should be undertaken to explore these issues fully.

Caution in interpreting some of the descriptive statistical findings is needed. For example, our mean DRS was 42.6, which is lower (better) than the average DRS scores found following the use of a FP decision aid in other studies (31). However, as some women reported using CFM after they had been referred to fertility services, it cannot be assumed that decisional regret outcomes can be solely attributed to our resource and adjustments to the woman’s diagnosis and its implications may have also influenced DRS scores.

There was little or no evidence in the individual patient case notes of women that may have been eligible to receive CFM, that a discussion regarding FP had occurred. We recommend that not only the FP discussion is documented but the quality and outcomes of any FP consultation should be recorded in the notes of cancer patients and reiterated in a summary clinical letter. Breast Cancer Now have developed a Fertility Toolkit for HCPs in breast cancer services which supports them to initiate and document a FP discussion with patients (47) but more work needs to be done to change practice and raise adoption of this tool or a local variation.

We found that CFM was generally used in the way it was intended by women, although some avoided reading some parts of the resource because of the emotions it invoked. This has been highlighted as a risk associated with FP patient decision aids (6), and other similar resources previously (48). It suggests that, given the benefits of such tools, patients should be encouraged to express any anxieties, concerns or other types of emotions e.g., guilt, during their clinical consultations that may arise from such interventions being used in routine care or research.

Despite the limitations and issues reported above, overall, our evidence suggests that CFM is a valid and acceptable resource to women with cancer facing the FP treatment decision. It better informed them about their FP options, enabling them to reason about the FP treatment decision in the context of their cancer treatment. It also supported conversations with others e.g., family members.

Our resource is intended to be used by women at risk of losing their fertility because of cancer treatment. The process of FP (information and possibilities) is deeply influenced by the disease site and its prognosis. In recognition of this, the resource reiterates the message that certain FP options may not be suitable or available to all women based upon their individual circumstances. But for many women, being fully informed about the full range of options and then understanding why some might not be suitable for them, we consider is an important part of the FP discussion. Our finding that all women found CFM better informed and supported them to make FP treatment decisions, regardless of the cancer type, supported our approach.

Since its development, it has also been converted into an online format, with both the print and digital formats freely available at https://cancerfertilityandme.org.uk. Its views online, adoption across a range of academic, clinical, policy and third-party sectors further demonstrates its value. At the time of writing there have been 20,902 page views of CFM online. Interestingly 60.4% are from the UK but the remainder from overseas visitors which may also suggest that the resource is having wider reach and interest internationally than previously anticipated. The resource has been endorsed by IPDAS, the UK National Institute for Health and Clinical Excellence (July 2020) and the UK government fertility regulator, the Human Fertilisation and Embryology Authority. A number of leading national cancer charities including Breast Cancer Now, Lymphoma Action, Brainstrust and Cancer Research UK all signpost patients to the CFM resource from their websites. Whilst the booklet version does not have to be read cover to cover, the one-page summary table, and the online version provide alternative versions of the resource for those concerned that the length of the booklet may limit its utilisation in clinical practice.

Our resource was initially informed by a systematic review of women’s values, treatment preferences and decision-making experiences (26), consideration of the evidence gathered from patients’ and clinician’s views on patient’s FP decision-making needs in the PreFer Study (27), a FP service evaluation of a local service (28) and an environmental scan and review of clinical guidelines (5, 6). Since our study started, more fertility preservation patient decision aids are now available to support women diagnosed with cancer around the globe (49). ESHRE have also recently produced a comprehensive guidance document which details and critiques the fertility preservation patient decision aids available (6). At least six are now intended for use by women without a certain cancer type (49). More recently, 24 innovative cancer-specific Dutch tailored patient decision aids have been developed to provide patients with personalised information which can be tailored to their cancer diagnosis and treatment (50). However, none of the existing tools have been co-developed and tested with women residing in the UK, and therefore CFM provides a valuable resource and addresses an unmet need for this patient group.

Our next goals are to evaluate the online version of CFM and undertake a randomised study to explore the effectiveness of the resource. Some effectiveness studies of the existing international FP patient decision aids are pending but the results from three (6), suggest that overall, they may better support women to make FP decisions. Except for one study finding slightly higher decisional conflict scores for those women that used their decision aid compared to usual care (32), the effectiveness studies found these interventions increased knowledge (31, 44), lowered decisional conflict (31, 32, 51), lowered decisional regret at 12 months (but only after adjusting for education) (31), improved patient satisfaction (31, 51) and reduced the time needed to make decision (51).

Despite the need for an effectiveness study, a recent study revealed that only 44% of patient decision aids were used in some capacity following their trial as a tool to support shared decision-making – key reasons for this were lack of funding, and disagreements between clinicians and patients over its use (52). A key goal for our team is to undertake an evaluation of the implementation and effectiveness of the resource, particularly amongst the breast and haematological cancer care pathways. Our findings show that the fertility HCPs seemed more comfortable in approaching patients and administering the resource compared with HCPs in cancer services. A new training package may prove helpful as part of a larger problem-solving approach to better support members of the cancer multidisciplinary teams to adopt CFM in their clinical practice and to have FP discussions that supports shared decision-making. Other approaches and models of care, such as multidisciplinary clinics integrating cancer and fertility joint working and service improvement developments to reduce the workload burden of cancer staff in busy clinics to meet patient need could also be considered. These issues were not the focus of our planned study but impacted and warrant further consideration for optimal patient care.

## Conclusion

In the context of a cancer diagnosis, the provision of our evidence-based CFM resource was successful in helping and better supporting women of reproductive age, to make FP decisions. However, whilst we addressed an unmet need for female cancer patients, at risk of losing their fertility, the research also highlighted some barriers which prevented access to use for these women at the time in the cancer pathway when they would have benefitted from it the most. Thus, using it as a tool to facilitate shared decision-making in oncology services requires further work. Given the challenges associated with patient decision aid recruitment, integration and adoption in routine clinical practice, existing frameworks could be modified to place a greater emphasis on identifying and addressing these issues during any need’s assessment work.

## Data Availability Statement

The anonymised raw data supporting the conclusions of this article may be made available by the authors upon reasonable request.

## Ethics Statement

NHS Ethics Approval was obtained from the Health Research Authority (Reference: 16/EM/0122) and we also obtained National Institute of Health Research (Clinical Research Network) portfolio status (CPMS ID 30522). All patients/ participants provided their written informed consent to participate in this study.

## Author Contributions

Principal investigator, conceived the idea (GJ), contributed to grant development and protocol (GJ, JH, DG, JG, GB-S, JS, and HB), contributed to the CFM steering group (GJ, RM, FD, NM, BP, JH, KV, DG, GB-S, JG, TC, DS, GV, JAS, EB, MM, DY, JS, SL, HB, RA), contributed to CFM patient decision aid development (GJ, RM, FD, NM, BP, JH, KV, DG, GB-S, JG, TC, DS, GV, JAS, EB, MM, JS, SL, HB, RA), assisted with patient and service user recruitment (RM, FD, NM, BP, JH, KV, DG, GB-S, DS, GV, JAS, EB, MM, DY, JS, RA), assisted with data collection and analysis (GJ, RM, FD, NM, JH, KV, RA), undertook the environmental scan (NM, HB, JH, GJ), assisted with the development of the online version of CFM, and the IPDAS and NICE endorsement submissions (GJ, RM, FD, RA), wrote the first draft of the manuscript (GJ, RM, FD, NM, KV and BP). All authors contributed to the subsequent drafts of the article and approved the submitted version.

## Funding

The study was funded by Yorkshire Cancer Research (S391).

## Conflict of Interest

The authors declare that the research was conducted in the absence of any commercial or financial relationships that could be construed as a potential conflict of interest.

## Publisher’s Note

All claims expressed in this article are solely those of the authors and do not necessarily represent those of their affiliated organizations, or those of the publisher, the editors and the reviewers. Any product that may be evaluated in this article, or claim that may be made by its manufacturer, is not guaranteed or endorsed by the publisher.
