# Supplementary material for: Cancer, Fertility and Me: Developing and Testing a Novel Fertility Preservation Patient Decision Aid to Support Women at Risk of Losing Their Fertility Because of Cancer Treatment
Source: Front Oncol. 2022 Jun 30;12:896939. doi: 10.3389/fonc.2022.896939 (PMC9280471; doi:10.3389/fonc.2022.896939)
Supplement: Supplementary file 1 [file DataSheet_1.docx]

**Supplementary Figure 1: Overview of study methods and total recruitment figures**

**
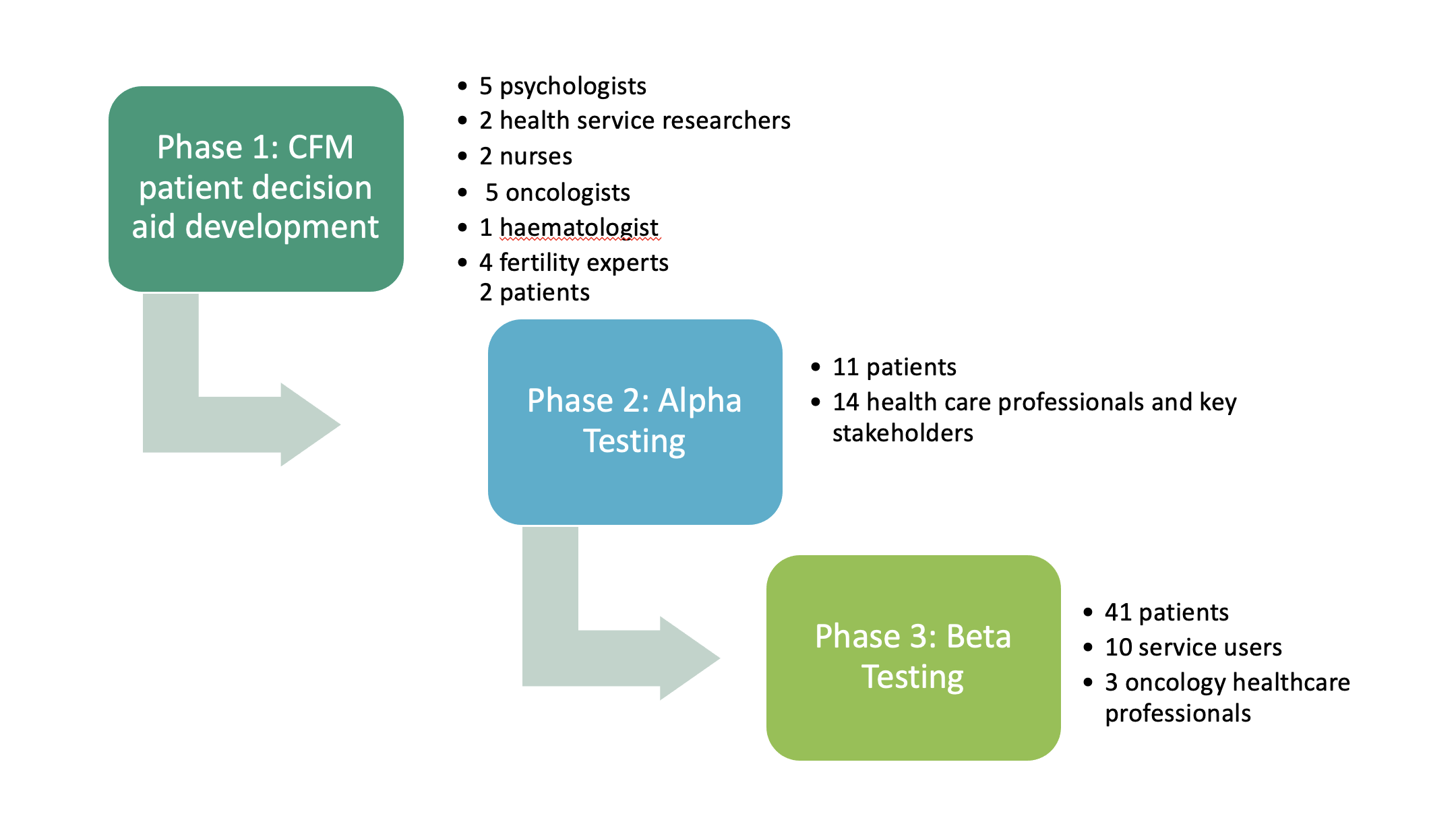
**

**Supplementary Figure 2a: Decisional Conflict Scale**

**
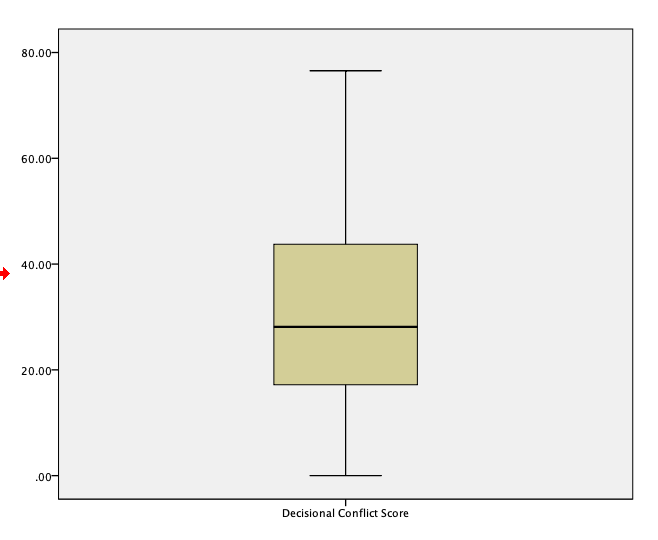
**

- Given at BASELINE ONLY
- Available for 37 women (missing = 4)
- Range 0: Low Decisional Conflict - 100: High Decisional Conflict
- No difference at baseline between those who were interviewed (mean: 30.8, SD: 22.0) and those who were not (mean: 30.7, SD: 21.4)

**Supplementary Figure 2b: Decisional Regret Scale**

**
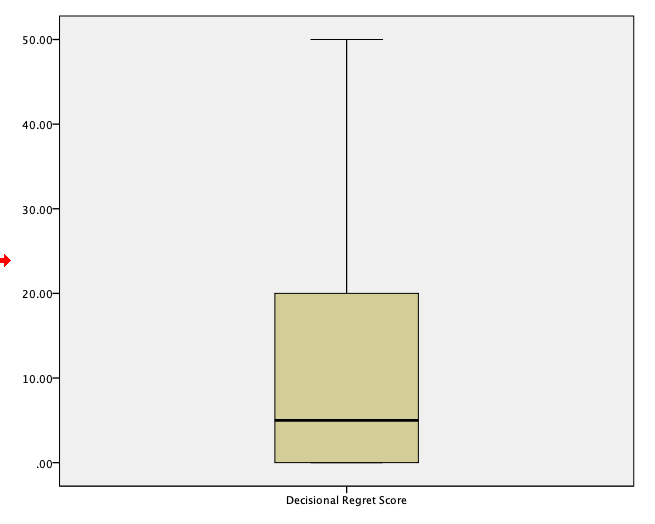
**

- Given at Time 4 only (at time of interview)
- Available for 29 women (missing =2)
- Range 0: Low Decisional Regret - 100: High Decisional Regret
- Mean: 12.2 (SD: 13.7)
- Low Decisional Regret Scores; Range (0-50)
